# Is a combined i.v. and enteral glutamine regimen superior to a single i.v. glutamine regimen in severe thoracic trauma?

**DOI:** 10.1186/cc12186

**Published:** 2013-03-19

**Authors:** D Pavelescu, I Grintescu, I Luca-Vasiliu, L Mirea

**Affiliations:** 1 Emergency Hospital Floreasca, Bucharest, Romania

## Introduction

Glutamine regulates many biological functions in preserving the cell, acts as a key respiratory fuel and nitrogen donor for rapidly dividing cells, and modulates the expression of many genes associated with metabolism, cell defences and repair, and cytokine production. In severe thoracic trauma, glutamine supplementation is essential because the body consumes more than it produces and glutamine effects become dependent on its route of delivery.

## Methods

Fifty-two patients 19 to 78 years old with surgery for severe thoracic trauma were assessed in two groups: Group A received 0.3 to 0.5 g/kg/day i.v. glutamine + 20 g enteral glutamine for 7 days, supplementation to enteral nutrition; Group B receive only i.v. glutamine supplementation to enteral nutrition 0.3 to 0.5 g/kg/day for 7 days. Weaning time, the duration of p.o. ileus, incidence and time to resolution of VAP, glycemic level and the percentage decrease of CRP at 96 hours were assessed in both groups.

## Results

**Figure 1:**
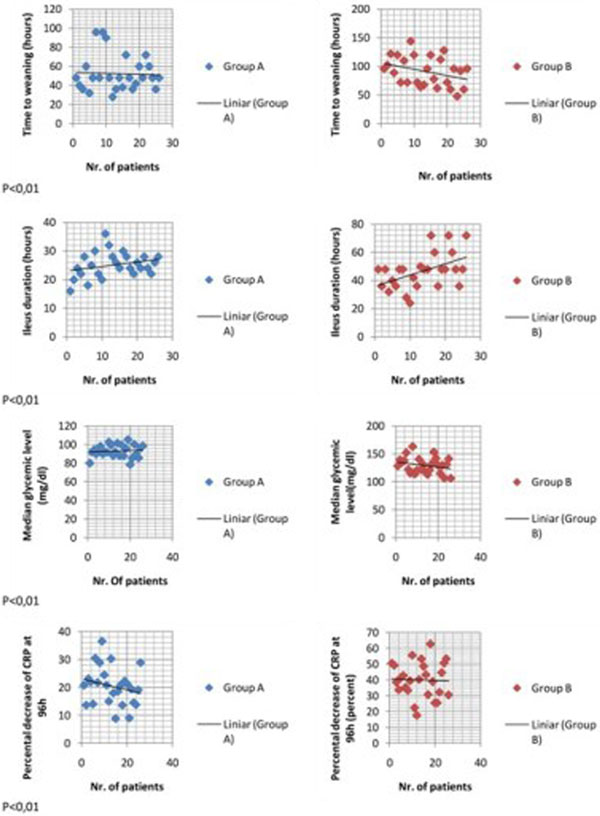
**Results**.

Weaning time and the duration of p.o. ileus were significantly lower in Group A; although the incidence of VAP is similar in both groups, the time of VAP resolution is lower, the glycemic control is better in Group A. The percentage of CRP decrease is higher in Group A. See Figure 1.

## Conclusion

Glutamine becomes an essential amino acid in severe thoracic trauma and when the patients are fed other than TPN (enteral, oral); although hard evidence is lacking, both administration routes may be efficient as soon as possible.

